# Concurrent chemotherapy (carboplatin, paclitaxel, etoposide) and involved-field radiotherapy in limited stage small cell lung cancer: a Dutch multicenter phase II study

**DOI:** 10.1038/sj.bjc.6602979

**Published:** 2006-02-07

**Authors:** P Baas, J S A Belderbos, S Senan, H B Kwa, A van Bochove, H van Tinteren, J A Burgers, J P van Meerbeeck

**Affiliations:** 1 Department of Thoracic Oncology, The Netherlands Cancer Institute, Plesmanlaan 121, 1066 CX, Amsterdam, The Netherlands; 2 Department of Radiation Oncology, The Netherlands Cancer Institute, Amsterdam, The Netherlands; 3 Rotterdam Oncological Thoracic Study group ROTS, Rotterdam, The Netherlands; 4 Department of Pulmonology, Onze Lieve Vrouwen Gasthuis, Amsterdam, The Netherlands; 5 Department of Medical Oncology ‘De Heel’ Hospital, Zaandam, The Netherlands; 6 Department of Biometrics, The Netherlands Cancer Institute, Amsterdam, The Netherlands

**Keywords:** SCLC, limited stage, concurrent radiotherapy, involved field

## Abstract

To improve the prognosis of limited stage small cell lung cancer (LS-SCLC) the addition of concurrent thoracic radiotherapy to a platinum-containing regimen is important. In the Netherlands, we initiated a multicenter, phase II study, of the combination of four cycles of carboplatin (AUC 5), paclitaxel (200 mg m^−2^) and etoposide (2 × 50 mg orally for 5 days) combined with 45 Gy (daily fractions of 1.8 Gy). The radiation was given to the involved field and concurrently with the second and third chemotherapy cycle. Patients with a partial or complete response received prophylactic cranial irradiation to a dose of 30 Gy. From January 1999 to December 2001, 37 of the 38 patients with LS-SCLC entered were eligible for toxicity analysis and response. Grade 3 and 4 haematological toxicity occurred in 57% (21/37) with febrile neutropenia in 24% (9/37). There were no treatment-related deaths or other grade 4 toxicity. Grade 3 toxicities were oesophagitis (27%), radiation pneumonitis (6%), anorexia (14%), nausea (16%), dyspnea (19%) and lethargy (22%). The objective response rate was 92% (95% confidence interval (CI) 80–98%) with a median survival time of 19.5 months (95% CI 12.8–29.2). The 1-, 2- and 5-year survival rate was 70, 47 and 27%, respectively. In field local recurrences occurred in six patients. Distant metastases were observed in 19 patients of which 13 in the brain. This study indicates that combination chemotherapy with concurrent involved-field radiation therapy is an effective treatment for LS-SCLC. Despite PCI, the brain remained the most important site of recurrence.

Small cell lung cancer (SCLC) accounts for approximately 15–20% of all lung cancers and first-line combination chemotherapy has led to an important improvement in response and survival. The addition of thoracic radiation therapy (TRT) and prophylactic cranial irradiation (PCI) has further improved the overall survival (Pignon *et al*, 1992; Warde and Payne, 1992). Despite the addition of sequential irradiation to the thorax, patients with limited stage (LS) of the disease and a good performance status do not surpass survival rates of 20% at 2 years (Johnson *et al*, 1990).

Besides the choice of the best available chemotherapy regimens, the optimal timing of radiation in relation to the chemotherapy has been a matter of debate for many years. There are several lines of evidence that early (concurrent) radiation is superior to late (sequential) administered radiation therapy (Murray *et al*, 1993). In Europe, the combination of cyclophosphamide, doxorubicin and etoposide has been used for many years but this regimen does not allow concurrent irradiation to the thorax due to the doxorubicin related risk of cardio pulmonary toxicity (Friedman *et al*, 1978; Torti *et al*, 1986).

Recently, several new drugs have been tested for their activity against SCLC and have shown promising results (Ettinger, 2001). Paclitaxel has demonstrated promising activity in extensive disease as a single agent with a response of 34–68% (Ettinger *et al*, 1995; Hainsworth and Greco, 1995) and has shown to act as a radiosensitizer (Kirkbride *et al*, 1997; Langer *et al*, 1997; Choy *et al*, 2000).

Carboplatin is considered to be nearly equivalent to cisplatin in efficacy in SCLC while offering the advantage of outpatient administration (Green and Seal, 1990; Skarlos *et al*, 1994). Like paclitaxel, carboplatin has radiosensitising properties, which makes the combination of the two drugs with radiotherapy interesting (Thomas *et al*, 1997).

Hainsworth *et al* (1997) have investigated the combination of carboplatin, paclitaxel and etoposide in a dose escalation study in 1997. Concurrent TRT was given during cycle 3 and 4 to patients with LS (45 Gy in 25 fractions) and was considered acceptable. Based on these results, we decided to perform a multicentre, feasibility/phase II study to evaluate the toxicity and efficacy of carboplatin, paclitaxel and etoposide combined with concurrent involved field TRT (starting concurrent with the second cycle) in chemo-naive patients with LS SCLC.

## PATIENTS AND METHODS

Eligible patients had biopsy or cytology proven LS SCLC. Chest X-ray, CT scan of the chest and upper abdomen, bone scintigraphy and MRI or CT-scan of the brain were standard examinations. No prior chemotherapy or radiation was allowed. Patients had to be older than 18 years, had to have a performance status WHO grade 0–2 and a measurable or evaluable lesion and no major organ failure, Haemoglobin >6.0 mmol l^−1^, WBC >3.0 × 10^9^ l^−1^ and platelets >100 × 10^9^ l^−1^, normal ASAT and ALAT (<2.5 × ULN), bilirubin (<2 × ULN), Lactate dehydrogenase within 1.5 times ULN, serum creatinine level <1.25 normal or creatinine clearance >60 ml min^−1^. Patients were exclude if there were signs of distant metastases or a weight loss of >10% in the preceding 3 months. Before registration, the radiation oncologist had to decide whether the tumour volume allowed the delivery of concurrent TRT. All patients gave written informed consent and the ethics committee of all participating hospitals approved the study.

### Chemotherapy

Patients were treated with four cycles of chemotherapy given every 3 weeks. This consisted of a three-drug combination. Paclitaxel (Bristol Myers- Squibb, Woerden, The Netherlands) was given at a dose of 200 mg m^−2^ in a 3 h intravenous infusion. Standard premedication consisted of dexamethasone, clemastine and ranitidine.

Carboplatin (Bristol Myers- Squibb, Woerden, The Netherlands) was given directly after administration of paclitaxel by a 30 min infusion. The dose was calculated using the Calvert formula (Calvert *et al*, 1989). Etoposide capsules (Bristol Myers- Squibb, Woerden, The Netherlands) were taken orally in a daily dose of 2 × 50 mg for 5 days following the infusions of carboplatin and paclitaxel. In case of vomiting, no redosing was allowed.

Dose adjustments or delays or were made when haematological toxicity occurred. Redosing was only permitted when the WBC was >3.0 × 10^9^ l^−1^, neutrophils >1.5 × 10^9^ l^−1^, platelets >100 × 10^9^ l^−1^ and no clinical signs of infection. If these conditions were not fulfilled the blood counts were repeated after 1 week. In case of a delay of more than 2 weeks, the patient went off study.

Dose reductions were applied for nadirs (platelets <50 × 10^9^ l^−1^; neutrophils <0.5 × 10^9^ l^−1^) with 25% reduction in carboplatin dose, 15% decrease for the paclitaxel dose and a 50% decrease in daily dose of etoposide.

For any nonhaematological grade 3 toxicity (except untreated nausea, vomiting and alopecia) the treatment was delayed until recovery.

When a patient experienced neurotoxicity CTC grade 2; the dose of paclitaxel was reduced with 15% and another 10% when the neurotoxicity persisted. When, despite dose reductions, grade 2 neurotoxicity persisted, the patient went of study.

Anti-emetics were given as prophylaxis according to the local practice. The use of growth factors was not allowed but prophylactic antibiotics could be used.

### Thoracic radiation therapy

**Figure 1:**
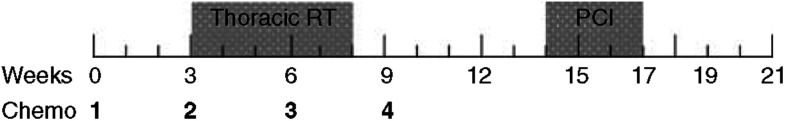
Time schedule of the four chemotherapy cycles, the radiotherapy treatment to the thorax and prophylactic cranial irradiation. The *X*-axis is in weeks. PCI: Prophylactic Cranial Irradiation.

A total dose of 45 Gy was given in 25 fractions of 1.8 Gy (five fractions per week) to the involved field. Thoracic radiation therapy started within 1 week after the start of the second cycle of chemotherapy (Figure 1). The target volume for irradiation was the primary tumour and all clinical and radiological involved lymph nodes with a short-axis diameter of ⩾1 cm (involved field irradiation). The mandatory radiotherapy planning CT scan, with intravenous contrast, was acquired shortly after the end of the first cycle of chemotherapy.

Megavoltage equipment was used with photon energies of 6 or 8 MV using a multileaf collimator or standard blocks to shape the irradiation portals according to the target volume. If possible anterior–posterior fields were applied. The radiotherapy treatment was interrupted for 1 week if the platelet count fell to ⩽30 × 10^9^ l^−1^ and if fewer than 20 fractions had been given. If thrombocytopenia occurred after more than 20 fractions had been given, the remaining fractions were omitted.

### Prophylactic cranial irradiation

Prophylactic cranial irradiation was given to patients with a complete or partial remission, starting five weeks after the end of last course of chemotherapy. A total dose of 30 Gy was administered in twelve fractions of 2.5 Gy each (four fractions a week), or 15 fractions of 2 Gy (five fractions a week).

### Baseline and response evaluation

Three weeks prior to the start of treatment, baseline tumour measurements were performed. This included a bronchoscopy, CT scan of the chest and upper abdomen and Chest X-ray. In all patients, a medical history, physical examination, performance status and laboratory values were assessed before start of treatment. Prior to every new cycle a medical history, weight, performance status, physical examination, complete blood count, liver and renal function tests were performed. The toxicity, occurrence of adverse effects, hospitalisations and use of concomitant medications was assessed prior to each cycle of chemotherapy. Toxicity was scored using the NCIC/CIC criteria (version 2.0, revised March 23, 1998). After completion of the treatment, patients were followed every 3 months until disease progression or death. A repeat bronchoscopy was performed in all responding patients when endobronchial tumour was observed at diagnosis. All radiological responses were confirmed by a second CT scan after 4 weeks. The time to progression was calculated from the end of treatment (excluding PCI) and the date of last follow-up or the date of disease progression, whichever happened first.

### Statistical analysis

This phase II study aimed to recruit a total of 50 patients in a 3-year period (EORTC, 1997). The study actually enrolled 38 patients in three years and it was decided to halt the trial because of the highly positive experience with this concurrent chemo-radiotherapy. This decision was also weighed by data from other studies (Kirschling *et al*, 1999; Thomas *et al*, 2001). Survival was analysed using a Kaplan Meier curve.

## RESULTS

### Patient characteristics

From January 1999 until December 2001, 38 patients were enrolled from eight centres. One patient did not start treatment because of uncontrollable hypertension and ECG abnormalities and was excluded from both toxicity and response evaluation. The ratio of male to female patients was 22 : 16 and all other patient characteristics are presented in Table 1. In all, 95% of patients had a performance status of 0 or 1. In 20 patients (52%) the primary tumour was located on the right side and there was involvement of the ipsilateral mediastinal lymph nodes in 26 patients (68%). Contra-lateral mediastinal lymph nodes were observed in seven patients (18%) and five patients (13%) presented with positive supraclavicular lymph nodes. In two patients, revision of the pathology revealed another type of tumour (one large cell neuro-endocrine tumour and one mixed small cell/nonsmall cell tumour). These patients, however, had already started the treatment and were include for the toxicity analyses.

### Dose administration and toxicity

For the 37 patients, 141 of the planned 148 cycles of chemotherapy were given. Five patients did not receive a fourth cycle and one patient stopped treatment after the first cycle because of disease progression. Adjustments of the chemotherapy schedule occurred in 62% (23/37) of the patients. In 12 out of the 37 patients (32%) a dose modification was given and in 14/37 (38%) a dose delay occurred. There were no treatment related deaths. Patients were hospitalised because of complications (febrile neutropenia, oesophagitis) in 15% and for logistical reasons in 25% (to appropriately co-ordinate the timing between chemotherapy administration and radiotherapy). The reasons for dose modification or delay are summarised in Table 2.

The highest toxicity recorded (grade 4) was haematological. Other grade 3 toxicity was lethargy (30%), oesophagitis (37%), dyspnea (26%), anorexia (19%), nausea and infection (22%) and vomiting (15%). Some possibly related neurotoxicity was observed (sensory, motor, vision, all 7%) (Table 3).

A total of 36 patients received TRT with a mean dose of 44.8 Gray. One patient was ineligible and one refused further therapy after the first cycle. Three patients missed 3 or 4 fractions due to oesophagitis complicated by fever, leucocyto- and thrombocytopenia or haemorrhage. In all, 30 patients were treated with PCI with a mean dose of 28.2 Gy. Toxicity related to the TRT was oesophagitis in 37% grade 3, 34% grade 2 and 37% grade 1. Nine patients (37%) required medical intervention (nasogastric feeding) for grade 3 oesophagitis. Oesophagitis grade 4 was not observed. Two patients required steroid treatment and oxygen administration for a grade 3 radiation pneumonitis.

### Response and time to progression

**Figure 2:**
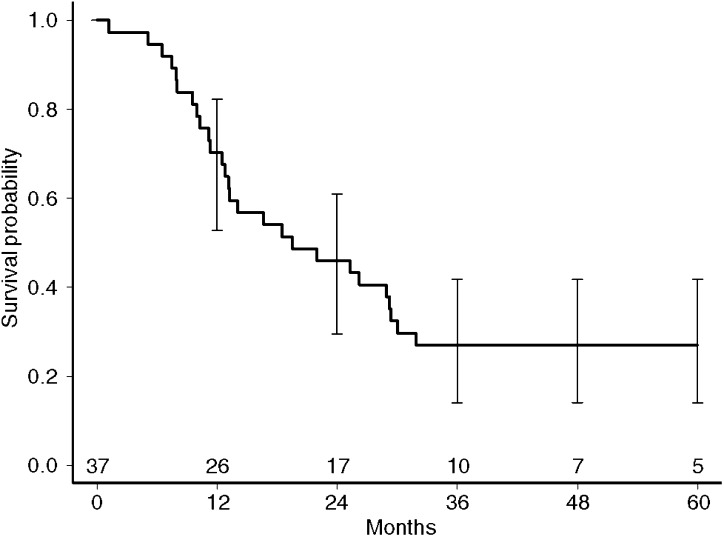
Kaplan–Meier survival curve. The *X*-axis shows the survival in months and the number of patients at risk. The survival probability is presented on the *Y*-axis.

An overall response of 92% (34/37 patients) was observed. In all, 16 patients achieved a complete radiological and histological response (CR), while 18 patients showed postradiation changes on the CT scan or chest X-ray while endobronchial examination revealed no residual tumour cells (PR). In three patients the response was not evaluable because of early toxicity. Figure 2 shows the overall survival curve, with a median overall survival of 19.5 months (95% CI 12.8–29.2 months). The 1-, 2- and 5-year survival rates were 70, 46 and 27%, respectively. The median time to progression (TTP) from start of chemotherapy for all patients was 15.9 months. Patients with a CR had 14.4 months TTP and for patients achieving a PR this was 23.9 months. At the time of analysis nine patients were still alive and progression-free.

Local recurrences (within the target area) were seen in six patients (16%) while distant recurrences occurred in 19 patients (51%). In 13 patients (35%) the first site of distant relapse was the brain.

## DISCUSSION

In this study, we have confirmed that the combination of carboplatin, paclitaxel and etoposide can be safely combined with concurrent involved field TRT. The overall response rate was 92%, which is comparable to other studies (Murray *et al*, 1993; Laurie *et al*, 2004). The 2- and 5-year survival rates of 46 and 27% are promising, especially when compared to the survival data of doxorubicine containing regimens with sequential radiotherapy (Bunn *et al*, 1986; Giaccone *et al*, 1993). Other platinum-containing regimens are considered more effective, even when combined with sequential radiotherapy as shown in an overview by Laurie *et al* (2004). This all adds to the evidence that concurrent chemo-radiation therapy should be considered standard treatment for patients with LS disease and good performance.

Overall, we observed a median TTP of 15.9 months. The difference in TTP between the patients with a PR and a CR may partly be explained by the definition of response. Owing to the concurrent use of chemotherapy and radiation to the thorax, there is an increased chance of pneumonitis and lung fibrosis, which will be visible on both chest X-ray and CT scan. This may also explain the relative high number of patients achieving a PR in our series. Turrisi (1998) reported similar data on the significance of ‘partial responses’ after concurrent platinum etoposide-TRT. In this intergroup study, 32% of patients were ‘partial responders’, and their 5-year survival after twice-daily radiotherapy was 23% while once-daily radiotherapy resulted in a 5-year survival of 8%.

This study shows that it is feasible to plan the chemotherapy and involved field TRT according to this protocol in a multicentre setting. Of all, 35 patients started the planned radiation therapy. The radiation therapy started within the first week of the second cycle, which allowed the radiation oncologist to optimally plan the radiation treatment and profit from possible tumour shrinkage after the first cycle. To prevent undesired interactions, the radiation dose on the first day of the third cycle was given before infusion of carboplatin and paclitaxel. Only three patients missed a limited number of fractions of radiation therapy due to haematological toxicity or oesophagitis. The mean total dose of radiation was 44.8 Gy with a significant but expected proportion of radiation oesophagitis (grade 3) of 37%. In two patients clinical and radiological signs of radiation pneumonitis occurred, requiring the use of oral steroids and oxygen support.

It is important to note that, despite PCI, 13 of the 30 patients presented with brain metastases as first site of failure. This is certainly an unexpected observation.

This is the first study prescribing involved field irradiation for LS-SCLC. The relatively low number of in field recurrences (six patients) supports the idea that concurrent TRT, with a reduced overall treatment time, has advantages over sequential TRT for loco-regional tumour control and is safe.

Concurrent thoracic radiotherapy seems to improve survival by eliminating chemo-resistant cells early in the treatment process. Although many studies have addressed this issue, the optimal timing of the radiation is not yet elucidated (Payne *et al*, 1994; Erridge and Murray, 2003). Takada *et al* (2002) reported a phase III study comparing concurrent and sequential radiation in combination with cisplatin and etoposide. The concurrent treatment arm had superior median survival (27.2 *vs* 19.7 months) and 5-year survival (23.7 *vs* 18.3%). Severe oesophagitis occurred more often in the concurrent treatment arm but was infrequent (<10%). Haematological toxicity (grade ⩾3) was 88% in the concurrent treatment arm and 54% in the sequential arm. This reported toxicity is comparable with our findings. Toxicity was predominantly haematological and resulted in a dose delay or reduction in 63% of the patients, which is considered an important issue.

The use of colony stimulating growth factors was prohibited because of the lack of reliable data and the possible detrimental effect when used with concurrent thoracic radiation. The SWOG performed a phase III study on the use of GM-CSF with concurrent chemo-radiotherapy (Bunn *et al*, 1995). The combination was associated with significantly deeper white blood cell and neutrophil nadirs, a significant increase in life-threatening thrombocytopaenia, longer hospital stay, higher incidence of intravenous antibiotic use, need for more transfusions, and a greater number of toxic deaths.

Another approach is the use of hyper-fractionated schedules in LS-SCLC. The effect of twice daily irradiation *vs* standard once daily radiation has been investigated by Turrisi *et al* (1999). This randomized trial compared once daily 1.8 Gy radiation *vs* twice daily 1.5 Gy radiation therapy to a total of 45 Gy. Both local control and survival were significantly improved in the experimental arm. The loco-regional recurrence rate dropped from 52 to 36% and at 5-year follow-up the survival was 16% in the standard arm compared with 26% in the experimental arm. This study has now led to new studies comparing high total doses of irradiation given once daily or by hyper fractionation. One of the disadvantages of hyper-fractionation is the increased risk of severe oesophagitis.

Recently, other chemotherapy regimens have been tested in SCLC. Two Japanese groups observed improved survival in patients with extensive stage (ES) SCLC when cisplatin was combined with irinotecan (Noda *et al*, 2002; Han *et al*, 2005). A median survival of 12.8 months for this new combination compared to 9.4 months for cisplatin/etoposide has attracted attention and this study is one of the few that have shown such a success for chemotherapy alone. In Europe and the USA, phase III studies are now underway to determine the exact role of irinotecan/cisplatin combination in ES SCLC. The preliminary results however do not confirm the Japanese findings. In addition, the implementation of irinotecan/cisplatin with concurrent TRT is quite complicated. Irinotecan is a potent radiosensitizer (Wu and Choy, 2002), it might, therefore, be expected that the combination of cisplatin and irinotecan with concurrent radiation therapy can lead to severe radiation induced oesophagitis and pneumonitis. So far the Japanese study by Han *et al* (2005) has not reported this.

The addition of a third drug in the treatment of SCLC has been questioned by at least two studies. The CALGB 9732 investigated the addition of paclitaxel to a combination of cisplatin and etoposide in patients with ES-SCLC (Neill *et al*, 2005). More toxic deaths (6.5 *vs* 2.4%) were reported in the three-arm combination and there was no improvement in overall survival. The RTOG 9606 study reported on the results of twice daily irradiation combined with paclitaxel, etoposide and cisplatin in patients with LS SCLC (Ettinger *et al*, 2005). In this phase II study, the radiation was given during the first cycle with a reduced dose of paclitaxel (from 175 to 135 mg m^−2^). The median survival of 24.7 months with a 54.7% 2-year survival rate is in line with other reported studies (Turrisi *et al*, 1999 and our study). Haematological toxicity (grade 3–4) occurred in 44% and oesophagitis grade 3 in 17% and grade 4 in 2% of the 53 evaluable patients. It can be concluded that for the treatment of both LS and ES SCLC a two-drug combination is probably sufficient.

Our study demonstrates that the three-drug combination and concurrent involved field thoracic radiotherapy is feasible in a multicentre setting in the Netherlands. The results also indicate that the haematological toxicity remains the major problem. Both survival and response rate seems superior to the combination of CDE and sequential radiotherapy. Nowadays our attention is directed to optimising the radiation schedules, delivering higher doses to the primary tumour and involved lymph nodes and to limit the haematological toxicity by using a two-drug regimen.

## Figures and Tables

**Table 1:** Patient characteristics

	**All**	
	***N***	**%**
All	38	100
		
*Gender*		
Male	22	58
Female	16	42
		
Age median (range)	65	(46–82 years)
		
*Smoking status*		
Never smoked	1	3
Smoked previously >10 years ago	6	16
Smoked previously ⩽10 years ago	31	82
		
*Active infection*		
No	37	97
Documented controlled	1	3
		
*Performance status*		
0	17	45
1	19	50
2	2	5
		
*Lymph node involvement*		
Ipsilateral mediastinal nodes	26	68
Contralateral mediastinal nodes	7	18
Supraclavicular nodes	5	13

One patient did not receive protocol treatment due to uncontrolled hypertension and ECG abnormality.

**Table 2:** Treatment details by chemotherapy cycle

	**Cycle number**			
	**1**	**2**	**3**	**4**
Number of cycles	37	36	36	32
				
Dose modification	1	2	7	12
				
*Reasons*				
Haematological toxicity		1	2	5
Other toxicity		1	5	7
				
Dose delay	1	3	13	14
				
*Reasons*				
Hyponatraemia	1			
Haematological toxicity		3	11	9
Other toxicity			2	5
				
Hospitalisation	9	11	15	7

**Table 3:** Toxicity according to the CTC criteria version 2.0/revised March 1998 for 37 patients

	**Grade 3**	**Grade 4**	**%**
*Haematological toxicity*			
Anemia	3	—	8
Neutropenia	13	8	57
Febrile neutropenia	9	—	24
Thrombocytopenia	5	3	14
			
*Non haematological toxicity*			
Anorexia	5	—	14
Oesophagitis	10	—	27
Diarrhoea	5	—	14
Nausea	6	—	17
Vomiting	4	—	11
Infection	6	—	17
Fever	1	—	3
Lethargy	8	—	22
Neurotoxicity	2	—	6
Dyspnea	7	—	19
Alopecia	2	—	6
Vision	2	—	6
Cardiovascular	3	—	8
Other (pain, hyponatremia)	5	—	14

The worst toxicity score (grade 3 and 4) per patient are presented.
